# Impact of Multidisciplinary Team Care on Patient-Reported Outcomes in Patients with Lung Cancer: A Systematic Review

**DOI:** 10.3390/curroncol32120697

**Published:** 2025-12-10

**Authors:** Aastha Srivastava, Elizabeth Daniel, Vincent Lam, Ru Karen Kwedza, Shelley Rushton, Ling Li

**Affiliations:** 1 Australian Institute of Health Innovation, Faculty of Medicine, Health and Human Sciences, Macquarie University, Sydney, NSW 2109, Australia; elizabeth.daniel@mq.edu.au (E.D.); vincent.lam@health.nsw.gov.au (V.L.); ru.kwedza@health.nsw.gov.au (R.K.K.); ling.li@mq.edu.au (L.L.); 2 Cancer Institute NSW, Sydney, NSW 2065, Australia; shelley.rushton@health.nsw.gov.au

**Keywords:** multidisciplinary team (MDT) care, lung cancer, quality of life (QoL), patient-reported outcomes (PRO), emotional well-being, physical well-being, social well-being, functional well-being

## Abstract

Background: Multidisciplinary team (MDT) care is now recognized as the most effective approach to managing lung cancer treatment. While MDTs aim to improve coordination, decision-making, and patient outcomes, their impact on patient-reported outcomes, particularly quality of life (QoL), remains unclear. Objective: This systematic review aimed to examine how the involvement of a multidisciplinary team (MDT) in the care of patients with lung cancer affects patient-reported outcomes and to investigate the enablers and barriers for implementing and running MDT care in lung cancer management. Methods: We systematically searched Medline, Embase, Cochrane, and Scopus (up to March 2024) to identify studies comparing QoL outcomes in patients with lung cancer managed with and without MDT care. The review was conducted and reported in accordance with the PRISMA 2020 guidelines. Risk of bias was assessed using the CASP tool, and findings were synthesized narratively. QoL outcomes were grouped into physical, functional, emotional, and social domains, and quantitative and qualitative data were synthesized narratively due to heterogeneity across studies. Results: Eleven studies met the inclusion criteria, comprising a total of 10,341 patients, with 3760 in MDT groups and 6581 in non-MDT groups. The methodological quality of the studies varied, with 10 papers rated as moderate to high quality. The findings suggest that MDT care may contribute positively to emotional support, and physical well-being. Better patient satisfaction and communication in MDT settings. Limitation: Heterogeneity and the lack of standardized PRO tools in outcome measures and study design limited comparability. Conclusions: MDT care may have a beneficial impact on certain aspects of quality of life in patients with lung cancer, particularly emotional and physical well-being. However, more robust and standardized research is needed to determine the full extent of its benefits on patient-reported outcomes.

## Simple Summary

Lung cancer is one of the deadliest cancers worldwide, and its care is complex, often requiring many healthcare professionals to work together. This systematic review examined how a multidiscipline team-based approach, where doctors, nurses, therapists, and other specialists collaborate on patient care, affects the lives of people with lung cancer. The researchers reviewed 11 international studies. These studies included over 10,000 patients with lung cancer. They found that patients cared for by teams generally experienced: better patient reported outcomes, such as physical health, less pain and fatigue, improved emotional well-being, and stronger social support. Patients also reported higher satisfaction with their care, feeling more informed and supported throughout treatment. The study identified factors that help or hinder this team-based care, such as early referrals, clear communication, and availability of resources. The findings show that coordinated, patient-focused care can improve quality of life for these patients. This highlights the importance of teamwork in healthcare. These insights can guide hospitals and healthcare systems to adopt more effective approaches, ultimately making cancer care more supportive, personalized, responsive and tailored to the needs of patients and their families.

## 1. Background

Lung cancer remains the leading cause of cancer-related deaths around the world. According to the World Health Organization (WHO), approximately 1.8 million deaths were reported in 2020 alone. This shows the urgent need for effective and coordinated treatment strategies [1]. The management of lung cancer is inherently complex. Therefore, it often requires quick decision-making and teamwork among different specialties to achieve the best patient outcomes.

In response to the complexities of cancer care, Multidisciplinary Teams (MDTs) have emerged as a central model of integrated, patient-centered care [2,3]. An MDT brings together healthcare professionals from various disciplines, like oncology, surgery, radiology, pathology, nursing, and allied health to work on personalized treatment plans for patients with cancer. This team-based approach ensures that all aspects of patient’s care are taken into account, promoting well-rounded and coordinated management [4,5]. Key characteristics of an effective MDT include teamwork, practical tasks, and flexibility, such as virtual MDT meetings initiated by the COVID-19 pandemic. This change suggests a new direction for cancer care [6].

Numerous studies and systematic reviews [7,8] have demonstrated that MDT involvement improves diagnostic accuracy, reduces time to treatment and results in better adherence to clinical guidelines [9]. As a result, MDTs are now seen as essential for high-quality cancer care and have quickly been adopted in health systems globally [10].

As the benefits of MDTs in improving survival rates and treatment planning are well-known, there is a growing need to understand their impact from the patient’s perspective. This is especially important for understanding patients’ quality of life and the overall care experience [6]. Patients with lung cancer often face significant physical, emotional, and psychosocial burdens, and understanding how multidisciplinary care affects these domains is critical for delivering truly patient-centered care [11,12]. Despite growing interest in patient-reported outcomes (PROs), there is limited evidence on how MDTs affect patients’ perspectives. This review will help us understand the impact of MDT on patient well-being, satisfaction, and quality of life. It will also guide the creation of more effective, patient-centered models of lung cancer care.

This systematic review aimed to

- To examine how the involvement of a multidisciplinary team (MDT) in the care of patients with lung cancer affects their overall quality of life, with a focus on understanding patients’ physical, emotional, social, and functional well-being through patient-reported outcomes (PROs).
- To investigate the enablers and barriers for implementing and running MDT care in lung cancer management. This included studying system-, process-, and patient-level factors that affect how MDTs function, communicate, and provide care.

By identifying these factors, the study will help us understand the challenges that might limit MDT effectiveness, and the key facilitators that promote successful, collaborative team-based care in lung cancer treatment settings.

## 2. Methods

### 2.1. Review Design

This systematic review was conducted in accordance with the Preferred Reporting Items for Systematic Reviews and Meta-Analyses (PRISMA 2020) guidelines [13], and was registered to Prospero with the record number of CRD42024591489. The PRISMA 2020 checklist was followed and is provided in Table S1.

### 2.2. Definitions and Scope of Multidisciplinary Team (MDT)

Lung cancer MDT is a group of healthcare professionals from different disciplines who work together to review and coordinate diagnosis, treatment, and supportive care. Core members include a medical or respiratory oncologist, thoracic surgeon, radiation oncologist (if radiotherapy is needed), radiologist, pathologist, and a specialist nurse or care coordinator [14]. Additional essential disciplines may include nuclear medicine and palliative care, depending on case complexity. Optional members often include physiotherapists, dietitians, occupational therapists, pharmacists, psychologists, social workers, general practitioners, and clinical-trials coordinators [14].

### 2.3. Search Strategies and Databases

The search strategy was developed based on the PICO (population, intervention, comparison, outcome) framework [15] to align with the review’s objective of evaluating the impact of multidisciplinary team (MDT) care on quality of life in patients with lung cancer (Table 1).

We searched the following databases: Medline, Embase, Cochrane Library, and Scopus. The search terms used for Medline are presented in Table 1. The final search was conducted on 13 August 2024, and detailed search strings are presented in Appendix A Table A1.

### 2.4. Inclusion and Exclusion Criteria

Studies were included if they involved patients diagnosed with any type and stage of lung cancer and if they examined the role of a multidisciplinary team (MDT) in patient management (Table 2). Studies had to describe MDT care models, such as tumor boards, coordinated care plans, or integrated approaches, and include a comparative group. Included studies were required to report outcomes related to quality of life, covering physical, emotional, social, and functional well-being, or patient-reported outcomes. Studies with appropriate designs, like randomized controlled trials, cohort studies, cross-sectional studies, and before–after studies, were considered. Studies that did not involve patients with lung cancer, lacked MDT care or a comparison group, did not report relevant outcomes, or used inappropriate designs, such as case reports, editorials, reviews, or non-peer-reviewed sources like conference abstracts, were excluded.

### 2.5. Study Selection

Three reviewers (A.S., I.L., E.D.) double-screened the titles and abstracts, and the full text screening was conducted. Any disagreement was first discussed between the reviewers. In the absence of a consensus, opinion was sought from a third reviewer (L.L.) for resolution.

### 2.6. Data Extraction

Two reviewers (A.S. and E.D.) conducted data extraction using a standardized form that included categories on (1) study characteristics, including study setting and design, (2) MDT definition and implementation, and (3) outcome of the intervention. The two reviewers, A.S. and E.D., met regularly to discuss and resolve any discrepancies or disagreements in data interpretation and data extraction of the studies. A third reviewer (L.L.) was consulted on the process and in case of disagreements. A chronicle synthesis of the findings was then conducted.

### 2.7. Critical Appraisal

The quality of included studies was assessed independently by two reviewers (A.S. and E.D.) using appropriate critical appraisal checklists [16]. Tools were selected based on study design, including checklists for RCTs, cohort studies, and pre–post intervention studies. Detailed assessments are presented in Appendix A Table A2.

### 2.8. Data Synthesis

A narrative synthesis of findings was conducted due to the heterogeneity in study design, outcome measures, and MDT models. Key findings were grouped according to dimensions of quality of life and patient-reported outcomes. Studies were grouped by study design, lung cancer stage, type of treatment, and PRO instrument. Within each comparison, the better-performing group was identified. Where available, published MCID thresholds were used to interpret clinical significance; for instruments without MCIDs, statistically significant improvements were interpreted as meaningful within the study context. Where applicable, barriers and enablers of MDT implementation were categorized using the Consolidated Framework for Implementation Research (CFIR) [17].

## 3. Results

**Figure 1:**
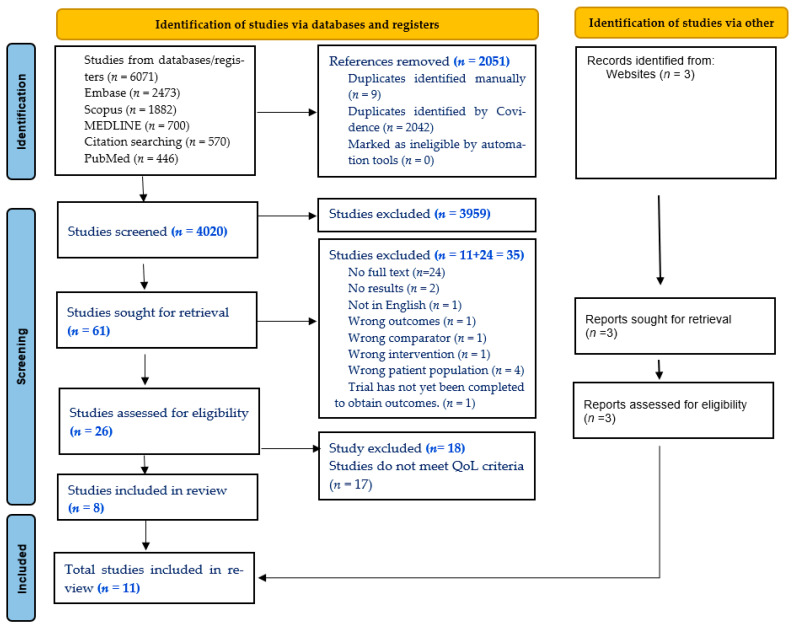
PRISMA flowchart.

A total of 6071 records were identified through database searching. After screening 4022 title and abstract, the full texts of 23 articles were obtained and assessed for final data extraction. Following eligibility screening, 9 articles met the eligibility criteria and an additional 3 articles were identified from other sources, bringing the total to 11 articles included in the review. We conducted a narrative synthesis of the findings (Table 3). Details of the studies screened and included at each stage are presented in the PRISMA flowchart in Figure 1. This review focuses on the outcomes of MDT versus non-MDT care and reports how these effects changed over time. The studies used validated PRO tools, but as the follow-up times and MDT structures differed, the results varied. MDTs worked best for symptoms like fatigue, pain, and anxiety. Patient satisfaction, as reported in one study, was very high. Several system, process, and patient factors helped or hindered MDT care. These factors explain why MDT outcomes differed across studies.

### 3.1. Study Characteristics

A narrative synthesis of the findings was conducted (Table 3). This review included data from eleven international studies investigating the impact of MDT care on patients with lung cancer, with a total of 10,341 patients: 3760 in MDT groups and 6581 in non-MDT groups. The proportion of male patients ranged from 33% to 70%, and the median or mean age spanned from 63 to 76 years across studies. Most studies focused on non-small cell lung cancer (NSCLC), with stages I to IV, and some emphasizing advanced or palliative care stages (IIIB–IV).

Study settings varied, including comprehensive cancer centers, community-based systems, and universities, across the USA (*n* = 5), Australia (2), Japan (1), China (*n* = 1), Denmark (*n* = 1), and Taiwan (*n* = 1). Most of the studies employed a randomized clinical trial (*n* = 6) and cohort study design (*n* = 3), followed by pre–post study designs (*n* = 2).

The composition and key characteristics of MDTs are summarized in Table 3. Non-randomized studies were at risk of confounding due to differences in stage mix, treatment intensity, and hospital volume. In pre–post studies (Borneman (2008) [18]; Smeltzer (2018) [27]), stage mix and treatment intensity were partially compared at baseline, with Smeltzer (2018) [27] adjusting for baseline PRO scores and surgical method using ANCOVA. Cohort studies (Raz (2016) [24]; Gregersen (2024) [23]; Shao (2023) [25]) either reported baseline similarities without formal adjustment Raz (2016) [24] or accounted for confounders via frequency matching, propensity score matching, or multivariable regression (Gregersen (2024) [23]; Shao (2023) [25]). RCTs and controlled clinical trials (Chen (2023) [19]; Edbrooke (2019) [20]; Ferrell (2015) [21]; Friedman (2016) [22]; Schofields (2013) [26]; Wang (2014) [28]) addressed key baseline differences through randomization or ANCOVA.

Follow-up times for PRO assessments ranged from 1 month to 12 months, and lack of standard 3- or 12-month follow-ups introduced heterogeneity (Borneman (2008) [18]; Chen (2023) [19]; Edbrooke (2019) [20]; Wang (2014) [28]). All studies used standardized, validated PRO instruments, though cultural adaptation was inconsistently reported.

### 3.2. Intervention Description

Across the reviewed studies, MDT compositions varied, but they consistently included core medical professionals [29] such as respiratory medicine, thoracic surgery, medical oncology, radiation oncology, pathology, radiology, a nurse specialist, and palliative care. Other team members [29] included nuclear medicine, social work, physiotherapy, psychiatry or psychology, dietetics, and occupational therapy. These roles were often included to support holistic care. Most teams met weekly, though some, like Chen (2023) [19], met monthly. Others, such as Gregersen (2024) [23], provided multidisciplinary input without a formal meeting structure. A summary of MDT composition is presented in Table 4.

### 3.3. Patient Reported Measures—Quality of Life

MDT care appears most effective in improving physical, functional, and emotional domains, with the greatest benefits observed for fatigue, pain, physical functioning, and anxiety/depression. Benefits for social well-being and overall QoL are more variable, reflecting heterogeneity in patient populations, team composition, and care context. These findings suggest that MDT care can enhance multiple aspects of QoL (Table 5). Detailed information for each study is provided in Appendix A, Table A3.

Physical well-being outcomes were reported better in MDT groups in ten studies (Table 5). Patients in MDT groups experienced less fatigue, pain, and dyspnea. They also showed improved mobility and nutritional status. For example, Shao (2023) [25] found improvements in fatigue and 6 min walk distance. Chen (2023) [19] reported reduced pain levels and improved nutritional status. However, some studies such as Gregersen (2024) [23] did not show meaningful differences in fatigue, insomnia, or appetite loss between MDT and non-MDT groups. In fact, symptoms such as dyspnea and diarrhea slightly worsened in the MDT group, though these differences were not statistically significant.

Functional well-being was generally improved with MDT care. Studies by Ferrell (2015) [21], Raz (2016) [24], and Schofields (2013) [26] showed significantly higher scores in functional domains for MDT patients. Notably, Raz (2016) [24] interpreted changes using minimal clinically important difference (MCID) thresholds, demonstrating that observed improvements were clinically meaningful. Edbrooke (2019) [20] also found modest but statistically significant improvements in physical and role functioning. However, Gregersen 2024 [23], again, showed no significant benefit in functional scores.

Emotional well-being improved with MDT care in several studies. Chen (2023) [19], Shao 2023 [25], and Raz 2016 [24] found lower anxiety and depression levels and higher emotional functioning scores in MDT patients. However, Edbrooke (2019) [20] and Gregersen (2024) [23] found no significant differences. An aspect of Gregersen’s study, “burden of illness,” was worse in the MDT group (*p* = 0.04).

Social well-being was generally better in MDT groups, with higher social or family support and fewer financial difficulties. Raz (2016) [24] and Ferrell (2015) [21] showed the most notable improvements. Still, Gregersen (2024) [23] reported no meaningful differences, and Smeltzer (2018) [27] observed slightly lower social well-being scores in the MDT group, though still statistically significant.

Overall quality of life was consistently higher in the MDT group. Chen (2023) [19], Ferrell (2015) [21], and Raz (2016) [24] reported higher QoL scores. However, Borneman (2008) [18] and Gregersen (2024) [23] found no statistically significant improvement in global QoL with MDT care.

### 3.4. Overall Patient Satisfaction

Only one study, Friedman 2016 [22], examined patient satisfaction with MDT care. Most patients reported very high satisfaction across several areas. Respect for patient questions was rated as “very good” by 82.2% of respondents, with 17.8% rating it as “good.” Similarly, 82.9% rated the clarity of their condition and treatment explanations as “very good,” while 15.4% rated it as “good.” Satisfaction with the time spent during consultations was rated “very good” by 69.0% and “good” by 29.3% of patients. Regarding the usefulness of written recommendations, 72.0% of patients rated it “very good” and 26.0% “good.” Finally, when asked about their likelihood of recommending the service, 80.9% indicated “very good” and 18.3% responded “good”.

### 3.5. Barriers and Enablers

**Figure 2:**
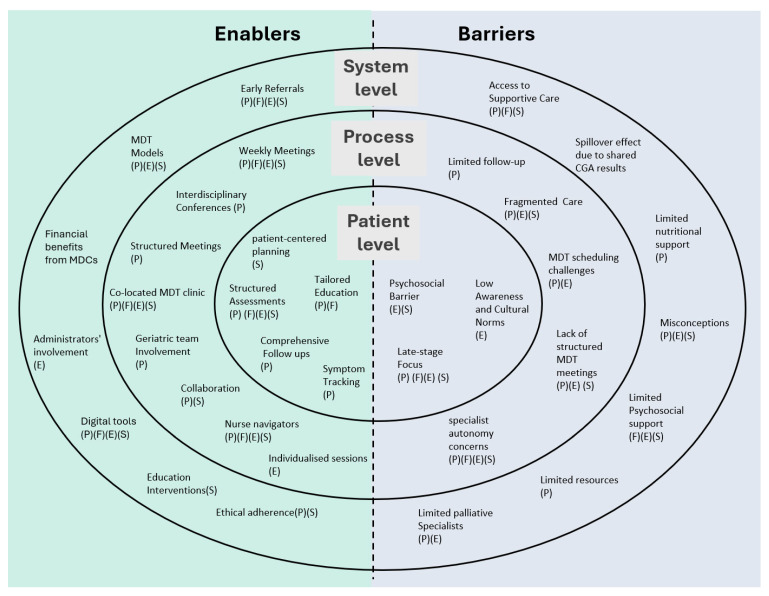
Enablers and barriers to the implementation of multidisciplinary team (MDT) care. Physical well-being (P), emotional well-being (E), social well-being (S), and functional well-being (F).

Across the system, process, and patient levels, several enablers and barriers influenced the implementation of MDT care (Figure 2). Enablers like early referrals, structured assessments, and co-located clinics support timely, coordinated, and patient-centered care. These factors improved overall well-being, including better symptom control, emotional support, social engagement, and functional status. In contrast, barriers like limited resources, fragmented care, and psychosocial issues reduce care effectiveness. They created delays, unmet needs, and a lower quality of life. The presence or absence of these factors significantly affected patient outcomes in MDT settings. Detailed study-level summaries are provided in Appendix A Table A4, Table A5, Table A6 and Table A7.

At the system level, enablers include early referral to palliative care and the use of standardized tools. Borneman (2008) [18] structured models like the E-Warm model. Chen (2023) [19] structured digital integration such as telehealth, whereas Raz (2016) [24] structured e-referrals. Financial benefits, including reduced redundant testing and improved cost-efficiency, also support system-level change (Friedman 2016) [22]. However, barriers persisted, such as limited healthcare and supportive care resources (Chen 2023 [19] and Ferrell 2015 [21]), a lack of palliative care specialists (Raz 2016 [24]), and infrastructural demands like significant scheduling changes for co-located MDT clinics (Smeltzer 2018 [27]).

At the process level, enablers include interdisciplinary case conferences, patient education interventions (Borneman, 2008 [18]), structured MDT meetings (Raz 2016 [24]), and the involvement of nurse navigators (Friedman 2016 and Smeltzer 2018 [22,27]). Multidisciplinary collaboration and tailored educational approaches also enhanced care processes as per Chen (2023) [19] and Ferrell (2015) [21]. Barriers, however included clinicians’ concerns over autonomy as highlighted in a study by Smeltzer (2018) [27]. Raz (2016) [24] showcased the lack of structured MDT meetings [24] and challenges in scheduling co-located services can hinder seamless integration.

At the patient level, education interventions and regular assessments are key enablers mentioned by Borneman (2008) [18] and Chen (2023) [19]. Barriers included psychosocial issues, cultural norms, and limited follow-up (Borneman 2008 [18] and Chen 2023 [19]) Tailored education faced challenges in late-stage care (Ferrell 2015 [21]) and fragmented care persisted in serial referral systems (Smeltzer 2018 [27]). Overall, these findings illustrated that while structured, multidisciplinary approaches can improve quality of life, implementing them needs to consider resource limitations, cultural factors, and structural fragmentation.

## 4. Discussion

In the 11 studies reviewed here, MDT care consistently showed improvements in physical functioning, emotional well-being, symptom burden, and overall QoL, highlighting the value of coordinated, multidisciplinary input for complex lung cancer care. These findings offer several implications for policy and service design. However, these benefits depend on how MDT interventions are structured and how often they occur. They are also affected by factors related to the healthcare system, the process, and the patients themselves. First, policymakers should prioritize investment in MDT models that embed routine symptom assessment, psychosocial support, and rehabilitation pathways, as these domains showed the strongest benefits. Second, effective MDT services require protected time, appropriate staffing, and clear clinical leadership to maintain continuity, communication, and timely decision-making. Lastly, adapting MDT care to resource-limited settings may require flexible strategies such as streamlined team composition, tele-MDT meetings, targeted training, and integration of community or primary care clinicians to ensure equitable access to multidisciplinary input even where specialist resources are constrained.

### 4.1. Multidisciplinary Team Composition and Process Characteristics

The variability in MDT composition and meeting formats observed in the included studies mirrors findings from the previous literature, such as Pillay (2016) [30]. This study highlighted that consistent team membership, strong leadership, and clear roles are vital for effective teamwork. In our review, we found that more structured and frequent MDT engagements (e.g., weekly formal meetings) were generally associated with better outcomes. This aligns with the findings of Prades (2015) [31], which emphasized the benefit of regular, protocol-driven interactions among core oncology professionals.

### 4.2. Physical and Functional Well-Being

Fatigue, dyspnea, pain, 6 min walk distance, and lung cancer physical subscale (e.g., Raz (2016) [24], Shao (2023) [25], Edbrooke (2019) [20]) show significant improvements in MDT patients (*p* < 0.05). Gregersen (2024) [23] shows mostly non-significant differences for fatigue, pain, insomnia, diarrhea, constipation, and mobility (*p* > 0.05), indicating minimal impact in these areas. Raz (2016) [24] and Ferrell (2015) [21] reported an association between MDT care and improved physical and functional well-being, primarily through better symptom control, streamlined care pathways, and proactive intervention. This correlates with the findings of Kochovska (2020) [32], who reported that MDTs facilitate earlier palliative care referral, which is associated with better pain management and symptom relief. Moreover, MDTs that integrate digital tools, as seen in Raz (2016) [24], echo the findings of Larson (2018) [33], who found that e-referrals and telehealth services reduce time to treatment and improve functional independence by minimizing delays in service delivery.

Conversely, where these system-level innovations were absent or inconsistently applied, such as in Gregersen (2024) [23] and Smeltzer (2018) [27], improvements in functional domains were negligible. This supports the assertion by Taplin (2015) [34] that MDT effectiveness is highly dependent on operational infrastructure, including data sharing systems and coordinated workflows.

### 4.3. Emotional Well-Being and Psychosocial Integration

Early emotional well-being measures Ferrell (2015) [21] show strong improvement (*p* < 0.001). Meanwhile, Gregersen (2024) [23] shows mostly non-significant changes for emotional functioning and future worries (*p* > 0.05).

The consistent emotional benefits observed in studies such as those by Chen (2023) [19] and Raz (2016) [24] underline the value of integrating psychological support within MDTs. This aligns with results from Jacobsen and Wagner (2012) [35], who found that oncology teams incorporating mental health specialists achieved better patient-reported outcomes on anxiety and depression scales. These findings reinforce the call for psychosocial oncology to be a standard element in MDTs, as advocated by the International Psycho-Oncology Society (IPOS) and endorsed in the 2018 ASCO guidelines [36].

However, studies like those by Smeltzer (2018) [27] and Borneman (2008) [18] caution that inadequate staffing or the lack of formal psychosocial protocols may negatively impact the effect of MDT care on emotional betterment. This aligns with the concept of “structural competency”—described by Metzl and Hansen (2014) [37]—which suggests that if patients’ mental health needs are not intentionally addressed, their emotional outcomes are likely to remain poor.

### 4.4. Social and Spiritual Well-Being

Gregersen (2024) [23] and Smeltzer (2018) [27] show no or negative effects on social functioning (*p* > 0.05). However, the findings regarding improved social and spiritual well-being in studies like those by Raz (2016) [24] and Ferrell (2015) [21] highlight the broader, holistic benefits of MDT care. These results are consistent with the work of Levit (2013) [38], who argue for the integration of supportive and spiritual care into oncology as a means of improving whole-person care. Tailored education and the inclusion of social workers and spiritual care professionals were important enablers in this context.

In contrast, when these roles were not clearly defined or underutilized, as seen in Borneman (2008) [18] or Gregersen (2024) [23], the social and spiritual domains of QoL either stagnated or declined. These outcomes highlight the need for equity-driven implementation of MDTs that address not just treatment needs but also the relational and existential challenges faced by patients with lung cancer.

### 4.5. Patient Satisfaction and Care Experience

Studies such as Friedman 2016 [22] mirror findings from O’Daniel (2008) [39], who demonstrated that patients report greater trust and clarity when decisions are made by a team rather than a single physician. This aligns with frameworks like the Picker Principles of Patient-Centered Care [40], which stressed the importance of communication, coordination, and emotional support as core to a positive care experience.

Nevertheless, satisfaction gains are dependent upon the visibility and coherence of MDT operations. Studies with poorly implemented MDTs (e.g., Gregersen 2024 [23]) often failed to show meaningful improvements, possibly due to patients not perceiving the benefits of multidisciplinary involvement if coordination is not evident in their care journey.

### 4.6. Other Barriers

The influence of barriers, such as limited staffing, resource constraints, and cultural barriers to care engagement, cannot be overstated. These issues were echoed in the studies by Borneman (2008) [18] and Smeltzer (2018) [27], and are consistent with global evidence, such as the findings by Andrulis (2007) [41], who emphasizes the need for cultural tailoring and health literacy-sensitive approaches to maximize MDT effectiveness.

### 4.7. Implications for Practice and Future Research

MDTs should adopt a more patient centric approach by ensuring that patients know who is involved in their care. This will encourage engagement, satisfaction, and trust. MDTs must be culturally responsive and fit the needs of different populations. Training in health literacy and cultural humility is crucial to reducing disparities and ensuring that everyone receives inclusive care. MDTs should include mental health professionals, social workers, and spiritual care providers to address the full range of patient needs. Additionally, using digital health tools like telehealth services, electronic referrals, and data systems can improve access to MDT care.

Evidence from the included studies indicates that the benefits of MDT care differ by patient subgroup. Early-stage patients gain most from MDT involvement, as it provides precise diagnostic assessment, staging, and treatment planning. In contrast, advanced-stage patients primarily gain from early supportive and palliative interventions. Meanwhile, patients with complex symptoms or psychosocial needs experience enhanced holistic care through the coordinated input of allied health and support services (Borneman 2008 [18]; Chen 2023 [19]; Edbrooke 2019 [20]; Ferrell 2015 [21]; Friedman 2016 [22]; Gregersen 2024 [23]). These findings highlight the importance of tailoring MDT involvement according to disease stage, symptom burden, and psychosocial complexity, providing practical guidance for clinicians in prioritizing MDT resources.

Future studies should use a long-term study design to evaluate the ongoing impact of MDT care on quality of life over time. There is also a need to assess how MDTs perform across diverse patient groups, including those who are underrepresented, culturally diverse, and socioeconomically disadvantaged.

## 5. Strength and Limitations

This review is the first to look at how multidisciplinary team (MDT) care affects patient-reported quality of life (QoL) in people with lung cancer. It also explores what helps and what hinders the effective use of MDTs. key strength is its structured methodology for the study design. It follows PRISMA standards and is supported by a thorough search strategy across major databases. The study uses established quality appraisal tools, CASP, to carefully evaluate the reliability and risk of bias in the included studies.

This review identified substantial variations among the included studies, with variations in study design, outcome measures, and the definition and implementation of MDT care. However, the heterogeneity across the studies hinders meaningful direct comparisons and precludes the possibility of conducting a meta-analysis. A key limitation of this review is the lack of standardized and validated tools for measuring quality of life in the included studies. Many studies relied on unidimensional or non-validated instruments, reducing the reliability and depth of the findings. Only peer-reviewed original research articles published in English were included to ensure accurate data extraction and interpretation, as language barriers could lead to misinterpretation of study methods, results, or outcomes. We acknowledge that this may introduce a potential language bias, which is a limitation of the review. However, studies from six different countries/regions are included in our review, including those where English is not the official language. These would help reduce bias from language and culture in our review. A further limitation is that few included studies adjusted for important demographic and social variables (e.g., age, employment, religion), reducing the ability to account for potential confounding.

To further explore potential reasons for non-significant findings, several factors—such as MDT maturity, resource limitations, and cultural context—may have influenced outcomes; however, these were not systematically examined or reported in the included studies. Future research investigating these factors could enhance our understanding of the variability in results. Where baseline characteristics were reported, we provide explanations accordingly, but firm conclusions cannot be drawn due to the lack of consistent reporting across studies. Additionally, the possibility of reverse causality contributing to non-significant findings is considered here for interpretative purposes, which remains within the scope of this review.

## 6. Conclusions

This review shows that MDT care benefits patients with lung cancer beyond survival. It improves physical, functional, emotional, and social well-being. Thus, MDTs support holistic, person-centered care and address complex patient needs across the disease trajectory. However, different Qo and measurement tools were used across included studies, which makes comparisons difficult and limits the generalizability. Therefore, standardized and validated instruments are beneficial for future research. Consistent reporting frameworks would also improve the quality of evidence. In the future, investment in strong MDT structures, combined with standardized patient-reported outcomes, will be essential for optimizing lung cancer care.

## Figures and Tables

**Table 1:** Search Strategy Based on the PICO (Population, Intervention, Comparison, Outcome) Framework.

PICO Concept Areas	MeSH Terms and Free Text Terms
Population (P): Patients diagnosed with lung cancer	“Lung Cancer” “lung neoplasm” “small lung cancer”
Intervention (I): Multidisciplinary team management	“Multidisciplinary team” “multidisciplinary team” “multidisciplinary care team” “tumor board” “multidisciplinary clinic” “multidisciplinary approach”
Comparison (C): Standard or non-multidisciplinary team management	N/A
Outcome (O): Effectiveness of treatment outcomes and survival rates, patient satisfaction	“Quality of Life”/exp OR “Patient-Reported Outcomes” OR “Patient Reported Outcome Measures” OR “Palliative Care”/exp OR “Symptom Management”

**Table 2:** Inclusion Criteria and Exclusion Criteria.

Category	Inclusion Criteria	Exclusion Criteria
Population	Patients diagnosed with any type of lung cancer (NSCLC, SCLC, and other lung neoplasms) at any disease stage (early, locally advanced, or metastatic).	Studies not including patients with lung cancer.
Intervention/Exposure	Involvement of a multidisciplinary team (MDT) in patient management, including roles such as oncologists, pulmonologists, palliative care specialists, nurses, and social workers.	Studies focusing solely on single-specialty care (e.g., surgery or chemotherapy only) or complementary/alternative medicine not integrated into MDT care.
Models of MDT Care	Studies describing different MDT care models (e.g., tumor boards, coordinated care plans, integrated care approaches).	Studies not describing or involving MDT-based care.
Comparative Group	Inclusion of a comparative group (e.g., historical or contemporaneous cohort without MDT care). Groups are inclusive of peri-operative care, systemic therapy, and palliative care pathways.	Studies lacking a comparative group (no control or comparison cohort).
Outcomes	Reporting on quality of life (physical well-being, emotional well-being, social well-being and functional well-being). Reporting on patient-reported outcomes (symptoms, side effects, emotional well-being, satisfaction with care).	Studies that do not report relevant outcomes related to MDT or QoL or Patient reported measures.
Study Design	Randomized controlled trials, controlled clinical trials, before–after studies, retrospective/prospective cohort studies, cross-sectional studies.	Case reports, editorials, opinion pieces, reviews, and studies without appropriate methodological design.
Language	Published in English.	Published in languages other than English.
Publication Type	Peer-reviewed original research articles.	Non-peer-reviewed publications, abstracts only, or conference posters without full data.

**Table 3:** Study Characteristics Table.

Author	Study Design Country	Setting	MDT Group (*n*) Non-MDT Group (*n*)	Male (%) Age (*y*)	Lung Cancer Type and Stage	Follow-Up Time Window	Quality of Study (Using CASP)
Borneman (2008) [18]	Pre-post study and Descriptive study United States	National Cancer Institute–designated Comprehensive Cancer Centre	Barrier Study—28 and 18 QoL Pilot-10	48% (Barriers Study), 33% (QoL Pilot) 64 (Barriers Study), 67 (QoL Pilot)	Stage I–IV	1 month 3 months	Moderate
Chen (2023) [19]	Randomized Controlled Trial China	Chongqing University Cancer Hospital	140 (Early palliative care group) 140 (Standard care group)	70% Mean 63	Stage IIIB-IV NSCLC	6 months	High
Edbrooke (2019) [20]	Randomized Controlled Trial Australia	Home-based rehabilitation	41 41	55% 72 years	Inoperable NSCLC and SCLC, mostly stage III–IV	9 weeks 6 months	Moderate
Ferrell (2015) [21]	Controlled Clinical Trial United States	California (outpatient thoracic surgery and medical oncology clinics)	272 219	38.5% <65 (46.4%), 65–74 (34%), ≥75 (19.6%)	NSCLC (Stages I–IV)	3 months 6 months 12 months	Low
Friedman (2016) [22]	Cohort Study United States	Lehigh Valley Health Network	52 57		Stage III NSCLC	No follow-up	Moderate
Gregersen (2024) [23]	Randomized Controlled Trial Denmark	Aarhus University Hospital (Oncological Outpatient Clinic)	182 181	55% Mean 76 (SD 4.6)	Cancer patients (prefrail and frail, non-surgical)	3 months	High
Raz (2016) [24]	Before–after (pre–post) study United States	National Cancer Institute-Designated Comprehensive Cancer Centre	38 33	42.40%	Not explicitly stated	6 months 12 months	High
Shao (2023) [25]	Randomized Controlled Trial Japan	Home-based care	36 35	76% 74	Inoperable NSCLC and SCLC; mainly Stage III–IV	3 months 6 months	High
Schofields (2013) [26]	Randomized Controlled Trial Australia	Multicenter (three oncology clinics in Victoria)	59 61	48.3% 66 years	Advanced (stage III/IV) (NSCLC)	8 weeks 12 weeks	Moderate
Smeltzer (2018) [27]	Cohort Study United States	Community-based healthcare system in Memphis, TN	178 348	50%	Various lung cancer stages, including Stage IV	3 months 6 months	Moderate
Wang (2014) [28]	Cohort Study Taiwan	National Health Insurance system	2724 5448	Not specified Mean 64.75	Newly diagnosed patients with lung cancer	3 months 6 months 12 months	Moderate

**Table 4:** MDT composition and meeting frequency.

Author	Core MDT Members	Allied Health/Support Members	MDT Meeting Frequency
Borneman (2008) [18]	Medicine, nurse specialists	Social work, chaplaincy, counseling, nursing assistants	Not reported
Chen (2023) [19]	Medical oncologists, oncology nurse specialists	Dietitians, psychologists	Monthly
Edbrooke (2019) [20]	Physiotherapist, Nurse	Occupational therapist, dietitian, and palliative care physician	8-week homebased program
Ferrell (2015) [21]	Oncologists, thoracic surgeons, nurse specialists, palliative physicians	Social workers, chaplains, dietitians, physical therapists	Weekly
Friedman (2016) [22]	Thoracic surgeons, medical and radiation oncologists, palliative care	Diagnostic radiology, pulmonary medicine, nutrition	Weekly
Gregersen (2024) [23]	Geriatricians, specialized nurses	Medication review, nutrition support, psychological support (via follow-ups, not formal MDT)	Not a formal MDT meeting
Raz (2016) [24]	Thoracic surgeons, nurse specialist, pulmonologists	Pain specialists, social workers, chaplains, dietitians, physical therapists	Weekly
Shao (2023) [25]	Physiotherapist	Dietitians, nurses, psychologists.	Delivered 8-week program: 2 home visits/week + 1 call/week
Schofields (2013) [26]	Oncologists, lung cancer nurse	Psychologists, Palliative care nurses	Weekly
Smeltzer (2018) [27]	Thoracic surgeon, medical oncologist, radiation oncologist, pulmonologist	Radiologist, nurse navigator	Weekly
Wang (2014) [28]	Physicians, nursing specialist	Psychological consultants, social workers, case managers	Not reported

**Table 5:** Summary of Patient-Reported Quality of Life Outcomes Across Domains (Physical, Functional, Emotional, Social, and Overall Well-Being).

Domain	Number of Studies Reporting Improvement	Findings
Physical well-being	8/10	Most studies report reduced fatigue, pain, dyspnea, and improved mobility/nutrition; some minor symptoms worsening in select studies.
Functional well-being	6/8	Improvements mostly in physical and role functioning; effect less consistent in one study.
Emotional well-being	7/9	Anxiety and depression reduced; some domains unchanged or worse (e.g., burden of illness).
Social well-being	5/7	Family support and financial outcomes improved; some studies showed minimal change or slight decrease.
Overall QoL	6/9	Higher total QoL scores in most studies; effect varies by patient group and study.

The studies included used various validated tools and scales to measure different aspects of quality of life (QoL). This approach showed the complex nature of patient-reported outcomes. The tools assessed areas such as physical, functional, emotional, and social well-being, as well as overall QoL.

## Data Availability

As this is a systematic review, no new data was created or analyzed. All data used in this study were derived from previously published studies, and relevant references are cited within the article.

## Supplementary Materials

The following supporting information can be downloaded at https://www.mdpi.com/article/10.3390/curroncol32120697/s1: Table S1: PRISMA_2020_checklist_Impact of MDT on PRO in Lung Cancer Patients SR [13].

## Appendix A

**Table A1:** Search strings.

Database	Search String
Medline	respiratory tract neoplasms/or exp lung neoplasms/or exp pleural neoplasms/or tracheal neoplasms/ ((lung or pleural or respiratory tract) adj2 (cancer * or neoplasm * or tumor * or tumor * or malignanc * or metast * or carcinoma *)). ti,ab. (lung cancer * or lung neoplasm * or non small cell lung carcinoma * or nonsmall cell carcinoma * or NSCLC or SCLC or small cell lung cancer *). ti,ab. or/1–3 Patient Care Team/, ti,ab. (multidisciplinary team * or MDT * or multidisciplinary care team * or multidisciplinary tumor board * or MTBs or multidisciplinary clinic* or multidisciplinary team meeting *). ti,ab. or/5–6 and/4, 7 limit 8 to english language
Embase	respiratory tract neoplasms/or exp lung neoplasms/or exp pleural neoplasms/or tracheal neoplasms/ ((lung or pleural or respiratory tract) adj2 (cancer * or neoplasm * or tumor * or tumour * or malignanc * or metast * or carcinoma *)). ti,ab. (lung cancer * or lung neoplasm * or non small cell lung carcinoma * or nonsmall cell carcinoma * or NSCLC or SCLC or small cell lung cancer *). ti,ab. or/1–3 Patient Care Team/, ti,ab. (multidisciplinary team * or MDT * or multidisciplinary care team * or multidisciplinary tumor board * or MTBs or multidisciplinary clinic * or multidisciplinary team meeting *). ti,ab. or/5–6 and/4, 7 limit 8 to english language
Cochrane	respiratory tract neoplasms or exp lung neoplasms or exp pleural neoplasms or tracheal neoplasms in Title Abstract Keyword OR (lung or pleural or respiratory tract) adj2 (cancer * or neoplasm * or tumor * or tumour * or malignanc * or metast * or carcinoma *) in Title Abstract Keyword OR (lung cancer * or lung neoplasm * or non small cell lung carcinoma * or nonsmall cell carcinoma * or NSCLC or SCLC or small cell lung cancer *) in Title Abstract Keyword AND Patient Care Team in Title Abstract Keyword OR (multidisciplinary team * or MDT * or multidisciplinary care team * or multidisciplinary tumor board * or MTBs or multidisciplinary clinic * or multidisciplinary team meeting *)
Scopus	(TITLE-ABS-KEY (“respiratory tract neoplasms”) OR TITLE-ABS-KEY (“lung neoplasms”) OR TITLE-ABS-KEY (“pleural neoplasms”) OR TITLE-ABS-KEY (“tracheal neoplasms”) OR TITLE-ABS-KEY ((lung OR pleural OR “respiratory tract”) W/2 (cancer * OR neoplasm * OR tumor * OR tumour * OR malignanc * OR metast * OR carcinoma *)) OR TITLE-ABS-KEY (“lung cancer *”) OR TITLE-ABS-KEY (“lung neoplasm *”) OR TITLE-ABS-KEY (“non small cell lung carcinoma *”) OR TITLE-ABS-KEY (“nonsmall cell carcinoma *”) OR TITLE-ABS-KEY (nsclc) OR TITLE-ABS-KEY (sclc) OR TITLE-ABS-KEY (“small cell lung cancer *”) AND TITLE-ABS-KEY (“patient care team”) OR TITLE-ABS-KEY (“multidisciplinary team *”) OR TITLE-ABS-KEY (mdt *) OR TITLE-ABS-KEY (“multidisciplinary care team *”) OR TITLE-ABS-KEY (“multidisciplinary tumor board *”) OR TITLE-ABS-KEY (mtbs) OR TITLE-ABS-KEY (“multidisciplinary clinic *”) OR TITLE-ABS-KEY (“multidisciplinary team meeting *”)) AND (LIMIT-TO (LANGUAGE, “english”))

The asterisk * () is a truncation symbol used in database searches to capture multiple word endings. For example, “cancer *” retrieves “cancer” and “cancers,” “tumor *” retrieves “tumor” and “tumors,” and “malignanc *” retrieves “malignancy” and “malignancies.” This ensures comprehensive retrieval of relevant studies.

**Table A2:** CASP result.

Author	Positive/Methodologically Sound	Negative/Relatively Poor Methodology	Unknowns
Borneman (2008) [18]	- Clearly focused research question - Appropriate cohort recruitment - Identified and accounted for confounders - Complete follow-up - Believable results aligned with existing evidence - Practice-relevant findings	- Insufficient detail on how exposure and outcome were measured - Short follow-up period - Lack of statistical precision reporting	- Applicability to local contexts unclear - Effect sizes and confidence intervals not reported
Chen (2023) [19]	- Clearly focused research question - Randomized controlled design - Clear intervention with comprehensive outcome assessment - Follow-up was complete and long enough - Results are believable and consistent with broader evidence - Highlights multiple outcome improvements, including survival	- No blinding, increasing risk of bias in self-reported outcomes - Did not identify or adjust for potential confounders - Single-center design limits generalizability	- No cost-effectiveness analysis - Limited detail on statistical adjustments - Applicability outside of China’s healthcare context is uncertain
Edbrooke (2019) [20]	- Clearly defined research question - Randomized controlled design - Intention-to-treat analysis - Balanced groups at baseline - Comprehensive outcome measures (physical, functional, QoL) - Reported precision (CIs, *p*-values) - Clinically relevant results - Relevant to community-based care settings	- No participant blinding - No investigator blinding	- Blinding of outcome assessors not stated - Long-term sustainability of effects unclear - Cost-effectiveness analysis not provided - Implementation feasibility in other health systems
Ferrell (2015) [21]	- The study clearly addressed a well-formulated research question. - All participants were accounted for at the study’s conclusion. - Effects of the intervention were reported comprehensively. - The study suggests that the experimental intervention may offer greater value than existing interventions.	- Randomization of participants to interventions was not performed. - There was no blinding of participants, investigators, or outcome assessors, introducing risk of bias. - The study groups were not similar at the start of the trial, which may affect comparability. - The precision of the treatment effect estimate was not clearly reported in one version. - Generalizability to the local population was unclear.	- Applicability to the local context is uncertain.
Friedman (2018) [22]	- The study addressed a clearly focused issue relevant to oncology care. - The cohort was recruited acceptably and both exposure (multidisciplinary care) and outcome measures were accurately recorded. - Follow-up was both complete and of adequate duration. - Results show a trend toward improved outcomes (e.g., 17 vs. 14-month survival), even if not statistically significant. - Findings align with other literature supporting the benefits of Multidisciplinary Clinics (MDCs), including better care coordination and adherence to guidelines. - Results are believable and applicable in contexts exploring MDC implementation.	- Important confounding factors were not adjusted for in the design or analysis. - The authors acknowledged but did not fully account for potential confounders, which limits the strength and generalizability of the results. - Precision of results is only described as “somewhat precise”; no clear statistical significance reported for survival benefit. - Applicability to local population is uncertain due to lack of adjustment for confounders	- It is unclear whether all confounding factors were identified. - Generalizability is only partially addressed
Gregersen (2024) [23]	- The study addressed a clearly formulated research question. - Participants were randomized, with stratified randomization used, strengthening internal validity. - All participants were accounted for at the conclusion of the study. - Outcome assessment was blinded at 90 days, helping reduce detection bias. - Validated quality of life (QoL) measures (EORTC QLQ-C30, QLQ-ELD-14) were used. - The study had a large sample size (363 patients), enhancing the reliability of findings. - The intervention was found to be potentially valuable compared to existing care. - Effects of the intervention were comprehensively reported, with good reporting on precision	- There was no blinding of participants or those delivering the intervention, introducing performance bias. - Heterogeneity in cancer types and patient frailty levels may have influenced outcomes, limiting internal consistency. - The study did not include a cost-effectiveness analysis, limiting its utility for economic evaluation or policy decision-making. - Applicability to local contexts and generalizability was marked as “can’t tell”, which reflects uncertainty in real-world translation.	- It is unclear whether the study groups were fully comparable at baseline
Raz (2016) [24]	- Clearly focused research question - Appropriate cohort recruitment - Exposure and outcomes accurately measured - Complete and adequate 12-month follow-up - Results are believable and practice-relevant - Fits well with existing literature	- Unclear whether confounders were fully identified and controlled.	- Applicability to local settings is uncertain - Limited reporting on precision (e.g., effect sizes/confidence intervals)
Shao (2023) [25]	- Clearly stated aim and research question - RCT design with randomization - Balanced baseline characteristics - Validated outcome measures (e.g., 6MWD, EORTC QLQ-C30, HADS) - High adherence and no adverse events - Comprehensive statistical analysis (*p*-values reported)	- No blinding of participants or investigators (open-label). - Potential for performance and detection bias. - Resource-intensive and not easily scalable.	- Whether outcome assessors were blinded - Long-term outcomes or sustainability not reported - Cost-effectiveness not assessed
Schofields (2013) [26]	- Clearly formulated research question - Randomization applied - Groups similar at baseline - Comprehensive outcome reporting - Precision of estimates reported - Equal treatment across groups - Intervention benefits outweigh harms - Applicable to local context	- No blinding of participants, investigators, or outcome assessors (open-label design). - Some risk of implementation bias due to non-blinded delivery.	- Not explicitly stated if outcome assessors were blinded - Unclear whether all potential confounders were fully controlled
Smeltzer (2018) [27]	- Exposure and outcome were accurately measured - Follow-up was complete - Findings are believable and likely applicable to other settings - Highlights implementation feasibility of MDT care	- No evidence that confounders were identified. - Unclear focus of research question.	- Recruitment process - Statistical precision and confidence intervals - Duration of follow-up - Comparison to other studies
Wang (2014) [28]	- The study clearly addressed a focused issue: The impact of Multidisciplinary Team (MDT) care on emergency department (ED) use among patients with lung cancer. - The cohort was recruited in an acceptable way, and both exposure (MDT participation) and outcomes (ED visits) were accurately measured, reducing risk of bias. - Follow-up was complete for the key outcome (ED visit rates). - The results showed an 11% reduction in ED visit rate for MDT participants, indicating a positive effect of coordinated care. - The results were believable and are consistent with other literature supporting MDT care in oncology. - The study implies that MDT care can improve healthcare effectiveness and may reduce avoidable ED visits.	- The authors did not clearly identify or adjust for all important confounding factors in the design or analysis, limiting internal validity. - The duration of follow-up and whether it was sufficient is unclear. - Applicability to local or broader populations was unclear.	- Identification and adjustment for confounding factors were both rated as “can’t tell.” - It is unclear whether the follow-up period was long enough to assess broader or long-term impacts. - Applicability to the local context was also uncertain.

**Table A3:** Patient-Reported Quality of Life: Physical, Functional, Emotional, Social, and Overall Well-Being.

Author		Scale		Non-MDT	MDT	*p* Value
Physical Well-Being (N = 10)						
Borneman (2008) [18]		Physical QOL		5.8 (SD = 2)	5.5 (SD = 1.5)	<0.003
Chen (2023) [19]		Nutritional Status No (0–1)—PG-SGA		30 (21.43%)	53 (37.86%)	0.007
		Nutritional Status Mild (2–8)—PG—SGA		78 (55.71%)	67 (47.86%)	0.007
		Nutritional Status Severe (≥9)—PG—SGA		32 (22.86%)	20 (14.29%)	0.007
		No Pain (0)—NRS		76 (54.29%)	53 (37.86%)	0.003
		Mild Pain (1–3)—NRS		52 (37.14%)	67 (47.86%)	0.003
		Moderate Pain (4–6)—NRS		12 (8.57%)	20 (14.29%)	0.003
		Severe Pain (7–10)—NRS		0	0	0.003
Edbrooke (2019) [20]		Fatigue—EORTC QLQ-C30		39.7	30.9	0.01
		Dyspnoea—EORTC QLQ-C30		29.7	20.5	0.03
Ferrell (2015) [21]		Physical well-being (Early)		19.5 ± 6.2	23.3 ± 3.3	0.004
		Physical well-being (Late)		21.2 ± 6.2	22.2 ± 4.9	0.004
		Physical well-being (Total)		20.3 ± 6.2	22.8 ± 4.2	0.004
Gregersen (2024) [23]		Fatigue—EORTC QLQ-C30		+26.8 ± 31.3	+30.0 ± 33.7	0.50
		Insomnia—EORTC QLQ-C30		−1.77 ± 38.0	−10.2 ± 42.1	0.89
		Pain—EORTC QLQ-C30		+2.27 ± 33.4	−1.74 ± 32.5	0.63
		Dyspnea—EORTC QLQ-C30		−6.82 ± 32.4	+0.75 ± 32.3	0.06
		Appetite Loss—EORTC QLQ-C30		−2.78 ± 46.6	+0.75 ± 47.1	0.40
		Diarrhea—EORTC QLQ-C30		−5.05 ± 35.1	+2.24 ± 27.2	0.06
		Constipation—EORTC QLQ-C30		−2.27 ± 25.1	−1.99 ± 28.8	0.82
		Nausea/Vomiting—EORTC QLQ-C30		−1.89 ± 25.9	+2.86 ± 27.0	0.40
		Mobility—EORTC QLQ-ELD14		+3.11 ± 22.7	+1.87 ± 20.1	0.69
		Joint Stiffness—EORTC QLQ-ELD14		+5.30 ± 31.9	+6.97 ± 32.7	0.69
Raz (2016) [24]		Lung Cancer Subscale		23.6 ± 4.2	29.4 ± 2.1	<0.001
		Physical Well-being		22.2 ± 4.1	25.2 ± 2.3	<0.001
Shao (2023) [25]		6-Minute Walk Distance (6MWD, m)		352 ± 84	393 ± 92	0.02
		Dyspnea (mMRC)		2.1 ± 1.0	1.6 ± 0.9	<0.01
		Fatigue (FAS)		22.5 ± 5.3	18.9 ± 5.1	<0.01
Schofields (2013) [26]		Physical Well-Being (FACT-L)		18.6 ± 5.5	21.2 ± 4.3	0.005
		Fatigue (Distress thermometer)		65.6% reported	45.8% reported	0.02
		Pain (Distress thermometer)		49.2% reported	28.8% reported	0.02
Smeltzer (2018) [27]		Physical Well-Being		24.9 ± 7.0	24.9 ± 7.6	<0.001
Wang (2014) [28]		Fever		23.97%	25.46%	-
		Dyspnea and Respiratory Abnormalities		13.53%	13.23%	-
		Chest Pain		10.20%	7.94%	-
		Abdominal Pain		9.39%	9.99%	-
		Dizziness and Fainting		7.50%	7.17%	-
		Nausea and Vomiting		7.00%	7.00%	-
		Malaise and Fatigue		4.24%	5.17%	-
		Hemoptysis		4.21%	3.29%	-
		Headache		2.66%	2.00%	-
		Cough		2.24%	1.06%	-
		Sleep disturbances		2.17%	1.70%	-
**Functional Well-Being (N = 8)**						
Borneman (2008) [18]		Functional Scale		not reported	21.1 (SD = 7.1)	
Edbrooke (2019) [20]		Physical Functioning—EORTC QLQ-C30		64.5	70.8	0.04
		Role Functioning—EORTC QLQ-C30		51.0	64.6	0.04
Ferrell (2015) [21]		Functional Well-Being (Early)		14.6 ± 4.9	19.5 ± 3.5	<0.001
		Functional Well-Being (Late)		17.5 ± 5.4	16.6 ± 6.0	<0.001
		Functional Well-Being (Total)		16.1 ± 5.4	18.0 ± 5.1	<0.001
Gregersen (2024) [23]		Role Functioning—EORTC QLQ-C30		−6.94 ± 49.0	−14.4 ± 46.1	0.21
		Physical Functioning—EORTC QLQ-C30		−9.82 ± 25.5	−9.20 ± 26.2	0.89
		Maintaining Purpose—EORTC QLQ-ELD14		+1.26 ± 29.8	+1.62 ± 29.1	0.75
Raz (2016) [24]		Functional Well-Being		14.4 ± 5.1	22.6 ± 2.6	<0.001
Shao (2023) [25]		EORTC QLQ-C30 Physical Function		61.2 ± 17.6	71.5 ± 15.9	<0.01
Schofields (2013) [26]		Functional Well-Being (FACT-L)		13.3 ± 6.3	16.4 ± 6.0	0.01
Smeltzer (2018) [27]		Functional Well-Being		22.0 ± 7	22.1 ± 7.3	<0.001
**Emotional Well-Being (N = 9)**						
Borneman (2008) [18]		Psychological QOL		4.5 (SD = 2.2)	4.7 (SD = 1.4)	
Chen (2023) [19]		Anxiety Subscale—(HADS-A)		2.66 ± 2.86	1.45 ± 2.86	<0.001
		Depression Subscale—(HADS-D)		3.54 ± 4.61	1.5 ± 2.05	<0.001
		Depression (0–4)—No		111 (79.29%)	127 (90.71%)	0.003
		Depression—Mild (5–9)		5 (3.57%)	12 (8.57%)	0.003
		Depression—Moderate (10–14)		5 (3.57%)	1 (0.71%)	0.003
		Depression—Moderately severe (15–19)		5 (3.57%)	1 (0.71%)	0.003
Edbrooke (2019) [20]		Emotional Functioning—EORTC QLQ-C30		74.1	76.4	0.42
Ferrell (2015) [21]		Emotional Well-Being (Early)		16.7 ± 4.8	20.8 ± 3.5	<0.001
		Emotional Well-Being (Late)		17.6 ± 4.9	19.0 ± 3.9	<0.001
		Emotional Well-Being (Total)		17.6 ± 4.9	19.9 ± 3.8	<0.001
Gregersen (2024) [23]		Emotional Functioning—EORTC QLQ-C30		+5.67 ± 18.3	+8.25 ± 22.6	0.26
		Future Worries—EORTC QLQ-ELD14		−10.9 ± 29.1	−12.3 ± 32.5	0.57
		Burden of Illness—EORTC QLQ-ELD14		−3.66 ± 35.5	+6.84 ± 35.4	0.04
Raz (2016) [24]		Emotional Well-Being (FACT-L)		19.4 ± 3.6	23.2 ± 1.8	<0.001
		Psychological Distress		4.0 ± 2.3	1.0 ± 1.4	<0.001
Shao (2023) [25]		Anxiety (HADS-A)		9.3 ± 3.7	6.7 ± 3.2	<0.01
				8.5 ± 3.9	6.2 ± 3.5	<0.01
Schofields (2013) [26]		Distress Score (NCCN DT)		4.5 ± 2.4	3.1 ± 2.6	0.01
		Anxiety (HADS)		7.7 ± 4.6	5.5 ± 3.7	0.01
		Depression (HADS)		5.7 ± 3.7	4.2 ± 3.6	0.04
Smeltzer (2018) [27]		Emotional well-being		24.0 ± 6	24.2 ± 6.0	<0.001
**Social Well-Being (N = 7)**						
Borneman (2008) [18]		Social QOL		4.9 (SD = 2.3)	5 (SD = 1.9)	-
		Spiritual QOL		6.3 (Sd = 2.1)	5.8 (Sd = 2.2)	-
Edbrooke (2019) [20]		Social Functioning—EORTC QLQ-C30		78.1	82.4	0.25
Ferrell (2015) [21]		Social/Family Well-Being (Early)		20.4 ± 6.9	24.5 ± 5.0	<0.001
		Social/Family Well-Being (Late)		24.1 ± 4.3	22.7 ± 6.5	<0.001
		Social/Family Well-Being (Total)		22.3 ± 6.0	23.6 ± 5.8,	<0.001
Gregersen (2024) [23]		Social Functioning—EORTC QLQ-C30		+2.75 ± 24.5, *p* = 0.25	−4.48 ± 28.9, *p* = 0.24	0.24
		Family Support—EORTC QLQ-C30		0.51 ± 41.0	+1.24 ± 36.2	0.44
		Financial Difficulties—EORTC QLQ-C30		+1.27 ± 11.3	+0.75 ± 7.61	0.62
Raz (2016) [24]		Social/Family Well-Being		19.1 ± 9.1	25.6 ± 3.6	<0.001
		FACIT-Spiritual Well-Being		32.7 ± 9.5	43.1 ± 6.8	<0.001
Schofields (2013) [26]		Social Well-Being (FACT-L)		17.0 ± 6.0	19.3 ± 5.7	0.04
Smeltzer (2018) [27]		Social Well-Being		18.3 ± 3.9	17.7 ± 5.2	<0.001
**Overall Quality of Life (N = 9)**						
Borneman (2008) [18]		Overall QOL (0–10)		5.0 ± 2.1	4.6 ± 2.1	0.53
Chen (2023) [19]		FACT-L Scale		111.66 ± 14.90	117.81 ± 11.15	<0.001
		Trial Outcome Index (TOI)		70.66 ± 11.35	75.62 ± 8.62	<0.001
		Lung Cancer Subscale		29.64 ± 3.94	30.90 ± 2.96	<0.003
Edbrooke (2019) [20]		Global QoL—EORTC QLQ-C30		62.4	67.1	
Ferrell (2015) [21]		Overall FACT-L Early		93.7 ± 20.6	115.4 ± 12.6	<0.001
		Overall FACT-L Late		105.3 ± 20.1	105.8 ± 18.8	<0.001
Gregersen (2024) [23]		Global QoL—EORTC QLQ-C30		+1.52 ± 23.8	+0.93 ± 23.7	0.57
Raz (2016) [24]		FACT-L Total Score (0–140)		98.7 ± 20.5	126.1 ± 8.2	<0.001
Shao (2023) [25]		Global QoL (EORTC QLQ-C30		51.7 ± 20.3	62.5 ± 19.8	<0.01
Schofields (2013) [26]		Total QoL Score (FACT-L Total)		83.3 ± 18.1	91.6 ± 15.5	0.01
Smeltzer (2018) [27]		Lung Cancer Scale		32.2 ± 5.8	31.9 ± 5.5	<0.001
Green represents Better in MDT.	White represents No Difference.	Red represents Non-MDT Better.				

**Table A4:** Physical Well-being Outcomes vs. Enablers and Barriers.

Study	Physical Well-Being Outcomes	Key Enablers	Key Barriers	Result
Raz (2016) [24]	↑ Physical well-being: 22.2 → 25.2 (*p* < 0.001)	Digital tools (e-referrals, telehealth), weekly MDTs, tailored education	Limited availability of palliative care specialists, lack of MDT structure	Better in MDT
Chen (2023) [19]	↑ Lung cancer subscale: 29.64 → 30.90 (*p* < 0.003)	E-Warm MDT model, regular assessments, multidisciplinary collaboration	Limited resources, low awareness, limited psych/nutritional support	Better in MDT
Edbrooke (2019) [20]	↓ Fatigue: –6.7 points (*p* = 0.03) ↓ Dyspnoea: –6.2 points (*p* = 0.03)	Tailored 8-week MDT rehab program, Home-based delivery improving accessibility, Multidisciplinary involvement, Structured and individualized exercise plans	Patient frailty (advanced stage lung cancer), variation in home environments and adherence, limited system capacity for coordinated home-based MDT services	Better in MDT
Ferrell (2015) [21]	↑ Physical well-being early: 19.5 → 23.3 (*p* = 0.004) ↑ Total: 20.3 → 22.8	Standardized assessments, structured meetings, tailored education	Late stage focus of palliative care	Better in MDT
Smeltzer (2018) [27]	↑ Physical well-being: 24.9 → 24.9 (*p* < 0.001)	Nurse navigator, co-located MDT clinic, outreach to underserved patients	Scheduling challenges, specialist autonomy concerns, fragmented care	Statistically better, not clinically
Borneman (2008) [18]	↓ Physical QOL: 5.8 → 5.5 (*p* < 0.003, lower = better)	Early referral, standardized tools, interdisciplinary case conferences	Misconceptions, psychosocial barriers, limited follow-up	Better in MDT
Gregersen (2024) [23]	No significant differences in physical metrics Pain: +2.27 → −1.74 (*p* = 0.63) Dyspnea: −6.82 → +0.75 (*p* = 0.06)	Randomized allocation, ethical adherence, geriatric MDT involvement	High attrition, clinicians not blinded	No meaningful difference
Shao (2023) [25]	↑ 6MWD: 352 → 393 m (*p* = 0.02); ↓ mMRC, ↓ Fatigue (*p* < 0.01)	Home-based MDT visits (physio, dietitian, nurse, psychologist), structured 8-week program, high adherence	Resource-intensive, coordination complexity, limited scalability in systems lacking home care infrastructure	Better in MDT
Schofields (2013) [26]	↑ FACT-L physical well-being: 18.6 → 21.2 (*p* = 0.005) ↓ Fatigue: 65.6% → 45.8% ↓ Pain: 49.2% → 28.8%	Structured distress assessment (NCCN tool), weekly MDTs, lung cancer nurse coordinators	Time constraints, variation in services across sites	Better in MDT
Wang (2014) [28]	No meaningful difference across symptoms	Large sample, symptom tracking [18]	No MDT intervention, limited detail on enablers/barriers	No difference

↑ indicates an increase or improvement in the outcome; ↓ indicates a decrease or reduction in the outcome.

**Table A5:** Functional Well-being Outcomes vs. Enablers and Barriers.

Study	Functional Outcome	Key Enablers	Key Barriers	Result
Raz (2016) [24]	+8.2 in functional well-being	Digital integration, weekly MDTs, tailored education	Non prominent	Better in MDT
Chen (2023) [19]	+6.15 in FACT-L	Structured MDT, regular assessments	Low awareness, limited psychological support	Better in MDT
Edbrooke (2019) [20]	↑ Physical Functioning: +6.0 points (*p* = 0.01)	Multidisciplinary team coordination (PT, nurse, OT, etc.), personalized activity and care plans, emphasis on functional goal setting, and home-based setting enabled engagement	Difficulty in standardizing functional interventions at home, long-term follow-up	Better in MDT
Ferrell (2015) [21]	+4.9 early, +1.9 total	Structured meetings, education	Late-stage focus	Better in MDT
Gregersen (2024) [23]	No significant change	Randomized design, questionnaires	High attrition, clinician blinding	No meaningful difference
Smeltzer (2018) [27]	+0.1	Nurse navigator, co-located clinic	Autonomy concerns, fragmented care	No meaningful difference
Shao (2023) [25]	↑ EORTC QLQ-C30 physical function: 61.2 → 71.5 (*p* < 0.01)	Structured 8-week home-based MDT care, high adherence, baseline ECOG 0 higher in MDT	Blinding not feasible, process complexity, scalability concerns	Better in MDT
Schofields (2013) [26]	↑ Functional well-being: 13.3 → 16.4 (*p* = 0.01)	Structured needs assessment (NCCN Distress Thermometer), nurse coordinator support, weekly MDTs	Time constraints, service variability across sites, occasional gaps in referrals	Better in MDT
Borneman (2008) [18]	21.1 (no comparator)	Early referral, education	Psychosocial barriers, limited follow-up	Unclear benefit

↑ indicates an increase or improvement in the outcome.

**Table A6:** Emotional Well-being Outcomes vs. Enablers and Barriers.

Study	Emotional Outcome	Key Enablers	Key Barriers	Result
Chen (2023) [19]	↓ Anxiety: 2.66 → 1.45 (*p* < 0.001) ↓ Depression: 3.54 → 1.5 ↑ Normal Mood Cases: 79.3% → 90.7%	Structured E-Warm model, regular assessments, collaborative MDT care	Low awareness, cultural norms, limited psychological support	Better in MDT
Edbrooke (2019) [20]	↑ Emotional Functioning: +3.2 points (*p* = 0.21) ↓ HADS Anxiety: –0.8 (*p* = 0.61) ↓ HADS Depression: –0.7 (*p* = 0.55)	Supportive care integrated into MDT model, home-based setting reduced travel stress	No formal psychological or psychiatric intervention included	Better in MDT
Ferrell (2015) [21]	↑ Emotional Well-being (Early): 16.7→20.8 ↑ Total: 17.6 → 19.9 (*p* < 0.001)	Tailored education, structured MDT meetings, standardized assessments	Late stage focus of care	Better in MDT
Raz (2016) [24]	↑ Emotional Well-being: 19.4 → 23.2 ↓ Distress: 4.0 → 1.0 (*p* < 0.001)	Digital tools, weekly MDT meetings, individualized sessions	Lack of structured MDTs, limited palliative specialists	Better in MDT
Smeltzer (2018) [27]	↑ Emotional well-being: 24.0 → 24.2 (*p* < 0.001)	Nurse navigator, co-located MDT clinic, admin support	Scheduling conflicts, autonomy concerns, fragmented referrals	Better in MDT
Borneman (2008) [18]	↑ Psychological QOL: 4.5 → 4.7	Early referral, interdisciplinary conferences, patient education	Misconceptions, psychosocial barriers, limited follow-up	Slight improvement
Gregersen (2024) [23]	Emotional Functioning: +5.67 → +8.25 (*p* = 0.26) Future Worries: ↓ (ns)	Randomized MDT allocation, ethical rigor, comprehensive measures	High attrition, clinicians not blinded	No meaningful difference
Shao (2023) [25]	↓ Anxiety (HADS-A): 9.3 → 6.7 (*p* < 0.01) ↓ Depression (HADS-D): 8.5 → 6.2 (*p* < 0.01)	Home-based MDT (including psychologist), structured 8-week intervention, regular monitoring	Emotional distress may hinder engagement; cognitive/functional burden; no long-term follow-up	Better in MDT
Schofields (2013) [26]	↓ Anxiety: 7.7 → 5.5 (*p* = 0.01) ↓ Depression: 5.7 → 4.2 (*p* = 0.04) ↓ Distress: 4.5 → 3.1 (*p* = 0.01)	Use of NCCN distress thermometer, lung cancer nurse coordinators, systematic psychosocial screening	Emotional readiness of patients, stigma around mental health, variable access to psychology services	Better in MDT

↑ indicates an increase or improvement in the outcome; ↓ indicates a decrease or reduction in the outcome.

**Table A7:** Social Well-being Outcomes vs. Enablers and Barriers.

Study	Social Outcome	Key Enablers	Key Barriers	Result
Edbrooke (2019) [20]	↑ Social Functioning: +3.4 points (*p* = 0.15)	Holistic MDT care, including psychosocial support; home-based setting enabled patients to remain connected with family and carers	Short duration (8 weeks) may not capture long-term social gains	Better In MDT
Ferrell (2015) [21]	↑ Social/Family Well-being (Early): 20.4 → 24.5 ↑ Total: 22.3 → 23.6 (*p* < 0.001)	Tailored education, structured MDTs, patient-centered planning	Late-stage care focus	Significantly better in MDT
Raz (2016) [24]	↑ Social Well-being: 19.1 → 25.6 ↑ Spiritual Well-being: 32.7 → 43.1 (*p* < 0.001)	Weekly MDTs, digital referrals, personalized sessions	Poor MDT structure, limited specialists	Significantly better
Borneman (2008) [18]	Social QOL: 4.9 → 5.0 Spiritual QOL: 6.3 → 5.8 (no significant difference)	Early palliative referral, case conferences, education	Misconceptions, psychosocial barriers	No meaningful difference
Gregersen (2024) [23]	↓ Social Functioning: +2.75 → −4.48 (*p* = 0.24) Family Support: ~no change (*p* = 0.44)	Comprehensive CGA, randomization, ethical oversight	High attrition, lack of blinding	No meaningful difference
Smeltzer (2018) [27]	↓ Social well-being: 18.3 → 17.7 (*p* < 0.001)	Co-located MDT clinic, nurse navigator	Specialist autonomy concerns, fragmented care	Non-MDT better
Chen (2023) [19]	Not reported	E-Warm model, structured assessments, collaborative MDTs	Low awareness, lack of psychosocial support	Not assessed
Shao (2023) [25]	↑ Satisfaction with care team: 13.99 → 15.97 (*p* < 0.01) ↑ Global QoL: 51.7 → 62.5 (*p* < 0.01)	Psychosocial support integrated in MDT (nurse, psychologist), high engagement, personalized approach	Resource-intensive, limited scalability, no long-term data on social participation	Better in MDT
Schofields (2013) [26]	↑ Social Well-being: 17.0 → 19.3 (*p* = 0.04)	Nurse coordination, structured psychosocial screening via MDT	Variable service availability across sites; patients may underreport needs due to stigma or stoicism	Better in MDT

↑ indicates an increase or improvement in the outcome; ↓ indicates a decrease or reduction in the outcome.
